# Modeling and Analyzing Stem-Cell Therapy toward Cancer: Evolutionary Game Theory Perspective

**Published:** 2020-01

**Authors:** Zahra VEISI, Heydar KHADEM, Samin RAVANSHADI

**Affiliations:** 1. Department of Electrical and Computer Engineering, Razi University, Kermanshah, Iran; 2. Department of Electronic and Electrical Engineering, University of Sheffield, Sheffield, United Kingdom

**Keywords:** Immunotherapy, Stem cells treatment, Evolutionary game theory, Replicator equations, Equilibrium points

## Abstract

**Background:**

Immunotherapy is a recently developed method of cancer therapy, aiming to strengthen a patient’s immune system in different ways to fight cancer. One of these ways is to add stem cells into the patient’s body.

**Methods:**

The study was conducted in Kermanshah, western Iran, 2016–2017. We first modeled the interaction between cancerous and healthy cells using the concept of evolutionary game theory. System dynamics were analyzed employing replicator equations and control theory notions. We categorized the system into separate cases based on the value of the parameters. For cases in which the system converged to undesired equilibrium points, “stem-cell injection” was employed as a therapeutic suggestion. The effect of stem cells on the model was considered by reforming the replicator equations as well as adding some new parameters to the system.

**Results:**

By adjusting stem cell-related parameters, the system converged to desired equilibrium points, i.e., points with no or a scanty level of cancerous cells. In addition to the theoretical analysis, our simulation results suggested solutions were effective in eliminating cancerous cells.

**Conclusion:**

This model could be applicable to different types of cancer, so we did not restrict it to a specific type of cancer. In fact, we were seeking a flexible mathematical framework that could cover different types of cancer by adjusting the system parameters.

## Introduction

Thousands of people are annual victims of cancer (1). The disease is caused by genetic and epigenetic alterations, which can disturb cells’ growth and death rates (2, 3). Cancerous cells have interaction with non-cancerous ones (4), so they can spread their symptoms in different parts of the body (5, 6). Innate and adaptive immune systems usually identify cancerous cells and kill them at the beginning of their emergence and prevent forming tumors (7). Besides, after forming a tumor, the cancer-immunity cycle can identify and attack it (8).

Immunotherapy has recently been proposed for disease therapy, especially cancer therapy (3, 9). This method is based on improving the immune system’s response to cure diseases (10). Although the exact effectiveness of the method is not proven yet (11), studies have predicted a new hope for an eventual remedy for cancer (12). There are more studies regarding this method (11).

Different aspects of cancer have been modeled using mathematical methods (13) including body response (14) and therapeutic techniques toward tumors; profits of radiation to improve the anti-cancer response (15, 16); activation of oncogenes (7, 17); and inactivation of tumor suppressor genes (18). The dynamics of targeted cancer therapy have been investigated using a mathematical model of somatic evolution (19). Bacteria and/or pro-inflammatory cytokine TNF in a set of established murine modes of cancer was presented in (20). An agent-based method of immune and epithelial cell interactions in breast lobular epithelium was developed to earn perspicacity towards the prognostic potential of inflammation (21). A mathematical model of vascularized tumor growth and the bifurcation analysis of the model’s dynamics was presented (22). A quantitative theory for tumor growth under angiogenic simulator/inhibitor control was presented assuming that this growth was controlled by the evolution of the vascular network that supplies oxygen and nutrients to tumour cells (23); later, some modifications of the model were posed and conditions that guarantee the eradication of the tumor during the remedy are derived (24). More cancer modeling by mathematical tools can be found in (25–28).

Various mathematical approaches, such as game theory, replicator equations, and differential equations, have been applied in cancer investigation (29–31). The emergence of cancer was studied considering the interaction between healthy and cancerous cells as an evolutionary game (7, 32). Models based on game theory were established that involve mutant cells, immune system cells, and medications as players of the game (32, 33). Game theory was employed to study the connection between the different types of cells in a tumor (34, 35). Metabolisms related to cancer were investigated through an evolutionary game perspective (36). Analyses based on game theory suggested therapeutic propositions (37, 38), and optimal strategy selection (10) to overcome cancer. Random interaction rates in the evolutionary game helped to reduce the fitness of cancerous cells in order to eradicate tumors (5, 39, 40).

In this study, an evolutionary game between cancerous and noncancerous cells was applied to study behaviors of the mutant and healthy cells. We do not know the values of the amounts of the game parameters as depended on the cells’ characteristics and the immune system response. We analyzed the system by categorizing it into different scenarios based on the amounts of its parameters. After that, we used replicator equations to analyses each scenario. For cases with undesirable outcomes, stem-cell injection was suggested as a therapeutic approach. The effect of added stem cells on the system was considered by reforming the replicator dynamics and adding some new parameters to the system. Finally, by adjusting the stem cell-related parameters, the game convergence changed appropriately.

## Methods

### Evolutionary Game Model of the Interaction between Healthy and Cancerous Cells

An evolutionary game is defined by a set of strategies that are the players of the game and a corresponding payoff matrix which shows how players receive benefits or lose costs in interaction with each other (32).

In our model, we considered the interactions between cancerous and healthy cells as an evolutionary game between them. Herewith our strategies were cancerous cells (*C*) and healthy cells (*H*). The payoff matrix of the game is shown in [1].

[1] $$\begin{array}{l} \begin{array}{ccc} & C & H \end{array}\\ \begin{array}{c} C\\ H \end{array}\left[\begin{array}{cc} a,a & b,c\\ c,b & d,d \end{array}\right] \end{array}$$

where parameter *a* is the payoff (whether benefit or cost) of the strategy of *C* in interaction with another *C*; *b* is the payoff of *C* when interacting with *H*; *c* is the payoff of *H* in competition with *C*, and *d* is the payoff of *H* in interaction with *H*. We believed the amounts of these parameters vary from patient to patient, depending on the characteristics of their immune system and their types of cancer, and etc. In this work, we analyzed the game for different amounts of its parameters, and so, the model could be used for different patients by finding respective parameters of the system for them.

### Studying Dynamics of the System using Replicator Equations

We applied replicator equations to describe the system dynamics. Considering *x* and *y* as the frequency of individuals adopting the strategies *C* and *H*, respectively, replicator equations are as follows (39, 40):

[2] $$\{\begin{array}{c} \dot{x}(t)=x(f_{C}-f_{\mathrm{CH}})\\ \dot{y}(t)=y(f_{H}-f_{\mathrm{CH}}) \end{array}$$

where:

[3] $$\{\begin{array}{l} f_{C}=\mathrm{xa}+\mathrm{yb}\\ f_{H}=\mathrm{xc}+\mathrm{yd}\\ f_{\mathrm{CH}}=xf_{C}+yf_{H} \end{array}$$

where *f_C_*, *f_H_* and *f_CH_* are the average fitness of healthy cells, cancerous cells, and the combination of both cells, respectively (7). It is understood from [2] that if the fitness of a strategy is more than the average fitness, the frequency of the strategy will increase and when the fitness of a strategy is less than average fitness, its frequency will decrease. Finally, the system will converge to a point where the fitness of both strategies are the same and are equal to the average fitness.

### Effect of Adding Stem Cells

We controlled the number of stem cells by looking at the ratio of cancerous cells and the existing amount of stem cells, i.e., the greater ratio of cancerous cells, the higher the requirement of stem cells, and the greater level of existing stem cells, the lower the requirement of stem cells. The injected stem cells reduce the ratio of cancerous cells to healthy cells. Considering these explanations, we reformed system dynamics as follows:

[4] $$\{\begin{array}{l} \dot{x}(t)=x(f_{C}-f_{\mathrm{CH}}-\mathrm{zw})\\ \dot{y}(t)=y(f_{H}-f_{\mathrm{CH}})+(1-y)\mathrm{zw}\\ z(t)=\mathrm{xj}-\mathrm{zl} \end{array}$$

where *z* is the frequency of stem cells, *j* shows the effect of the ratio of cancerous cells on the ratio of stem cells, *l* indicates the impact of previously injected stem cells on the frequency of stem cells injected, and *w* shows how much the injected stem cells can reduce the frequency of the cancerous cells and increase the proportion of the healthy cells.

It could be understood from [4] that when the ratio of stem cells increases, the frequency of cancerous cells (healthy cells) will decrease (increase). Moreover, as the level of cancerous cells goes up, the ratio of stem cells should increase; as the proportion of stem cells increases the level of injection should decrease. The goal is to eradicate cancerous cells using stem cell injection which means to lower parameter *x* to a minimum level by adjusting stem cell-related parameters (*z*, *w*, *j* and *l*).

In order to facilitate the interpretation of the model, system parameters’ definitions are summarized in Table 1.

**Table 1:** Summary of the model parameters and other signs

***Parameter***	***Function***	***Parameter***	***Function***
*C*	Cancerous cell	*H*	Healthy cell
*x*	Ratio of *C*	*y*	Ratio of *H*
*z*	Ratio of stem cells	*a*	Payoff of *C* interacting with *C*
*b*	Payoff of *C* interacting with *H*	*c*	Payoff of *H* interacting with *C*
*d*	Payoff of *H* interacting with another *H*	*f_C_*	Average fitness of *C*
*f_H_*	Average fitness of *H*	*f_CH_*	Average fitness of *C* and *H* together
*W*	Effect of *z* on ***ẋ*** and ***ẏ***	*j*	Effect of *x* on ***ż***
*L*	Effect of *z* on ***ż***		

## Analysis

In this part, we analyzed the convergence of the system before and after the injection of stem cells. For cases that the convergence of the system without the injection of the stem cells was not desired, based on [2], we considered the effect of stem cell therapy on convergence of the model.

### Before Adding Stem Cell

Considering [2]; after some algebra analysis, given in the appendix, equilibrium point(s) of the system can be found as follows (As *x* + *y* = *1*, there is no need to consider parameter y in our analysis so we just consider x in our analyses).

[5] $$\dot{x}=0\Rightarrow (t_{1}+t_{2})x_{\mathrm{eq}}^{3}-(t_{1}+2t_{2})x_{\mathrm{eq}}^{2}+t_{2}x_{\mathrm{eq}}=0$$

[6] $$\text{then},x_{\mathrm{eq}1}=0,x_{\mathrm{eq}2}=1,x_{\mathrm{eq}3}=\frac{t_{2}}{t_{1}+t_{2}}$$

[7] $$\text{where},\;\;t_{1}=c-a,\;\;t_{2}=b-d$$

Both t1 and t2 are presented just to simplify the mathematical symbols in the paper and have no biological concepts. We categorized the system into different cases, as shown in the Table 2, to analyze its dynamics. Although the feasible interval for *x* was [0, 1], we investigated *x* even outside this interval because it could help to understand the system trajectories easily, discussed later.

**Table 2:** Different cases of the system

***Case name***	***Case feature***	***Sub-case name***	***Sub-case feature***	***Location of xeq3***
*A*	*t_1_+t_2_ > 0*	*A_1_*	*t_1_ < 0, t_2_ > 0*	1 < *x_3_*
		*A_2_*	*t_1_ > 0, t_2_ < 0*	*x_3_ < 0*
		*A_3_*	*t_1_ > 0, t_2_ > 0*	*0 < x_3_ < 1*
*B*	*t_1_+t_2_ < 0*	*B_1_*	*t_1_ > 0, t_2_ < 0*	*x_3_<0*
		*B_2_*	*t_1_ < 0, t_2_ > 0*	*1 < x_3_*
		*B_3_*	*t_1_ < 0, t_2_ > 0*	*0 < x_3_ < 1*

**Fig. 1:**
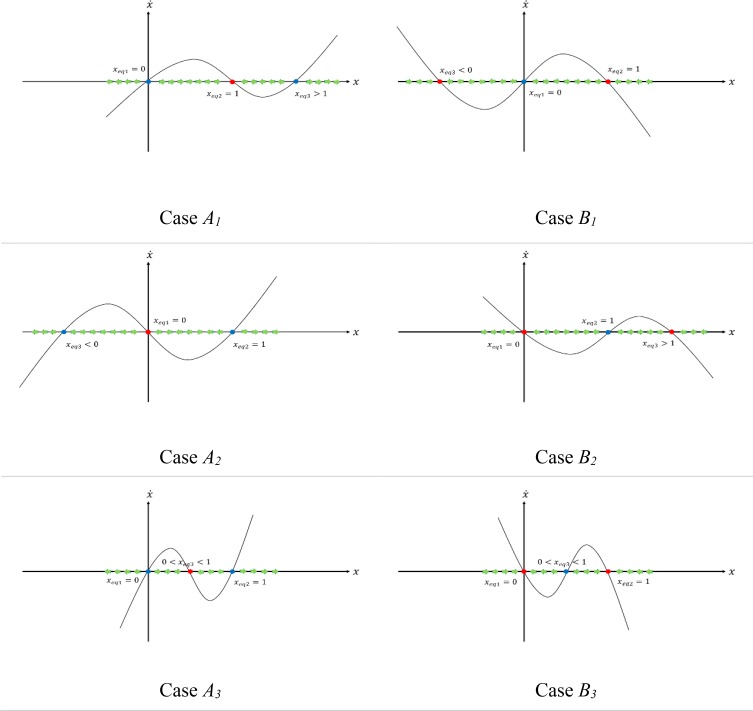
Convergence of the system in different cases

Fig. 1 shows the equilibrium points and trajectories of the system in different cases. In this figure, red and blue points indicate stable and unstable equilibrium points, respectively. Attraction and repulsion manifolds of equilibrium points are shown using green arrows.

The goal was that the system would converge to the points with no or a minimum level of cancerous cells, i.e., the whole interval (0, 1) become an attraction manifold for a stable equilibrium point located at *x = 0* or *x = 0^+^*. The parameter *x* in cases *A_2_* and *B_2_* converged to zero so these cases do not need extra analysis. However, we should have changed the trajectories of the model in other cases. Therefore, we adjusted the stem cell-related parameters (*j*, *w*, *l*) to change the trajectories, rather than setting the game parameters *a*, *b*, *c*, *d* which is an alternative method (Fig. 1).

### After Adding Stem Cells

By adding stem cells, based on [4] , the equilibrium points of the system changed as follows:

[8] $$\{\begin{array}{l} \dot{z}=0\Rightarrow z_{\mathrm{eq}}=\frac{j}{l}x_{\mathrm{eq}}\\ \dot{x}=0\Rightarrow (t_{1}+t_{2})x_{\mathrm{eq}}^{3}-(t_{1}+2t_{2})x_{\mathrm{eq}}^{2}+t_{2}x_{\mathrm{eq}}-wx_{\mathrm{eq}}z_{\mathrm{eq}}=0 \end{array}$$

then,

[9] $$x_{\mathrm{eq}1}=0,(t_{1}+t_{2})x_{\mathrm{eq}2,3}^{2}-(t_{1}+2t_{2}+e)x_{\mathrm{eq}2,3}+t_{2}=0$$

[10] $$\text{where},e=\frac{\mathrm{wj}}{l}$$

Like *t_1_* and *t_2_*, e is presented just to simplify the mathematical symbols in the paper and have no biological concepts.

Therefore, *x_eq1_* = 0 was still an equilibrium point, and its stability or instability was not affected, because ẍ (0) = *t*_2_ and the parameter *t_2_* has not changed (from control theory notions, we know that the sign of ẍ represents the stability or instability status of an equilibrium point).

The product and sum of the roots of [4] and [9], (*P_1_*, S_1_) and (*P_2_*, S_2_) respectively, are:

[11] $$\{\begin{array}{l} P_{1}=\frac{t_{2}}{t_{1}+t_{2}},\;S_{1}=\frac{t_{1}+2t_{2}}{t_{1}+t_{2}}\\ P_{2}=\frac{t_{2}}{t_{1}+t_{2}},\;S_{2}=\frac{t_{1}+2t_{2}}{t_{1}+t_{2}}+\frac{e}{t_{1}+t_{2}} \end{array}$$

Hence, the product of the roots was not affected after appearing the stem cell-related parameters in the system, so we used this fact in the later analyses.

Our strategy to converge the system to desired points in cases *A_1_*, *A_3_*, *B_1_*, and *B_3_* was as below.

First scenario: If *x_eq1_ = 1* was a stable equilibrium point (case *B_3_*), we made the whole interval (0, 1) an attraction manifold for this equilibrium point. Second scenario: If *x_eq1_ = 0* was an unstable equilibrium point (cases *A_1_*, *A_3_*, *B_1_*), we embedded a stable equilibrium point at *x = 0^+^* and then made the interval (0^+^, 1) an attraction manifold for this equilibrium point. We analyzed different cases based on our strategy.

### Cases *A_1_* and *A_3_*

**Fig. 2:**
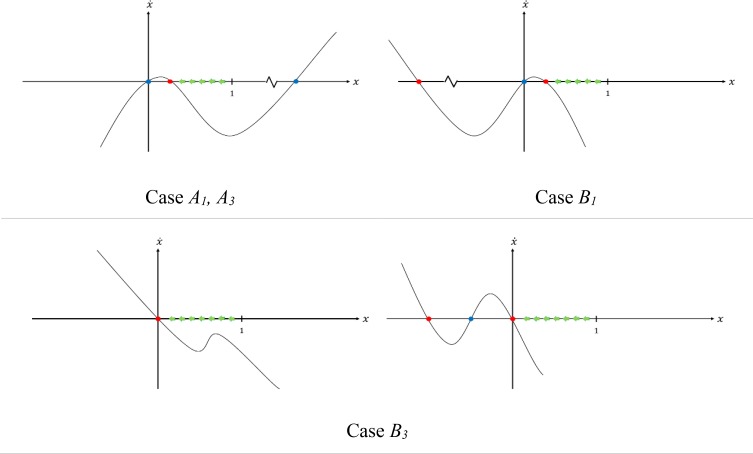
Reformed trajectories of the system after applying our aropositions

According to [11], by choosing positive values for *e*, the sum of the roots increase but the product remains constant. Thus the smaller root is decreasing and the bigger one is increasing (Fig. 1). By selecting *e = +∞*, the roots would be: *x_eq2_ =* 0^*+*^ (stable) and *x_eq3_ = +∞* (unstable), and the entire interval (0^+^, 1) would be an attraction manifold of *x_eq2_ = 0^+^* (Fig. 2).

### Case *B_1_*

In this case, for negative amounts of *e*, according to [10], the sum of roots would decrease and the product of roots is not changed. Therefore, both positive and negative roots shown in Fig. 1 would decrease, but their sign would not change. Hence, by *e = −∞*, the roots would be: *x_eq2_ = 0^+^* (stable) and *x_eq3_ = −∞* (stable), and the interval (0^+^, 1) stands as an attraction manifold for *x_eq2_ = 0^+^* (Fig. 2).

### Case *B_3_*

In this case, our strategy was based on the first scenario. We have shown in the appendix that by applying the condition suggested in [12], [9] has no root(s) in the interval (0, 1). Therefore, the whole interval would be an attraction manifold for *x_eq1_=0*. Fig. 2 summarizes the results of our analysis.

[12] $$-2{\left(t_{2}(t_{1}+t_{2})\right)}^{0.5}-(t_{1}+2t_{2})<e$$

## Simulations

Simulation results were presented to confirm the effectiveness of our propositions. In this section, without loss of generality, we assumed that *j = 1* and *l = 1*, *e = w*. Hence, it could be inferred from [8], the value of *z_eq_* depends only on *x_eq_*.

**Fig. 3:**
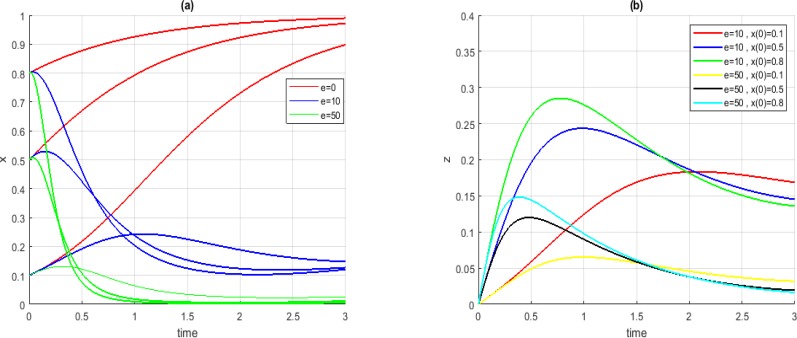
Convergence of *x* (a) and *z* (b) in Case *A_1_*, for *t_1_ = −1* and *t_2_ = +2*, different initial conditions and different values for *e*

Fig. 3(*a*) illustrates the convergence of *x* in Case *A_1_* for three different initial conditions before and after the injection stem-cells; Fig. 3(*b*) illustrates stem-cell injection level proposed by our approach for each prospective case shown in Fig. 3(*a*). In all cases, when the value of parameter *e* was 0, the parameter *x* converged to 1 not desired. By increasing the parameter *e*, the system converged to points with lower amounts for parameter *x*.

**Fig. 4:**
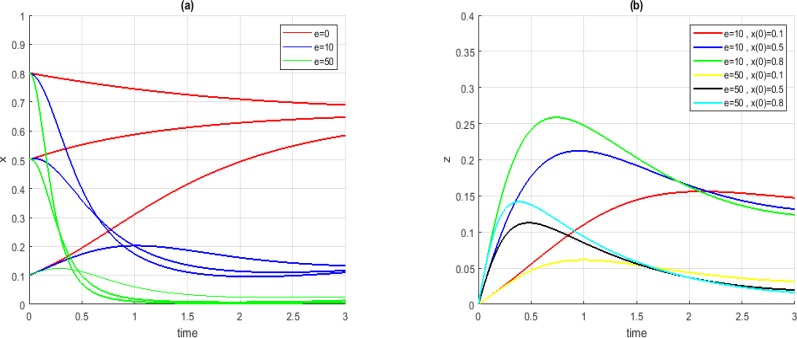
Convergence of *x* (a) and z (b) in Case *A_3_*, for *t_1_ = −1* and *t_2_ = +2*, different initial conditions and different values for *e*

Fig. 4 shows the convergence of the system and the level of stem-cell injection in Case *A_3_*. The results of this case is comparable to that of Case *A_3_*. As figure shows, following our suggestions, the system converged to points with lower levels of cancerous cells.

**Fig. 5:**
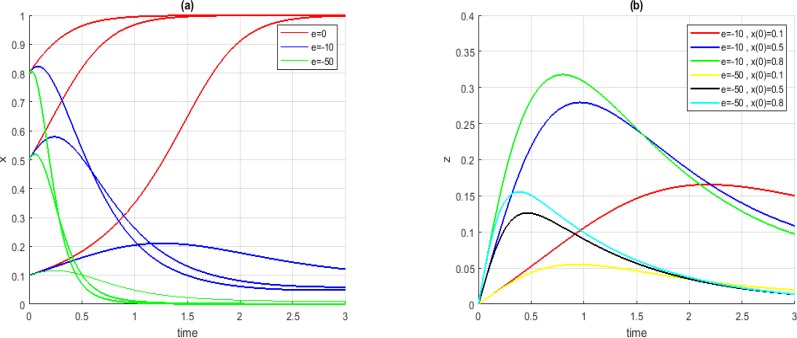
Convergence of *x* (a) and *z* (b) in Case *B_1_*, for *t_1_ =−1* and *t_2_ =+2*, different initial conditions and different values for *e*

The convergence of the system and the level of stem-cells in Case *B_1_* are presented in Fig. 5. For all initial conditions, before applying our propositions, the system converged to undesired points. However, by applying the suggestions the system converged to more desired points.

**Fig. 6:**
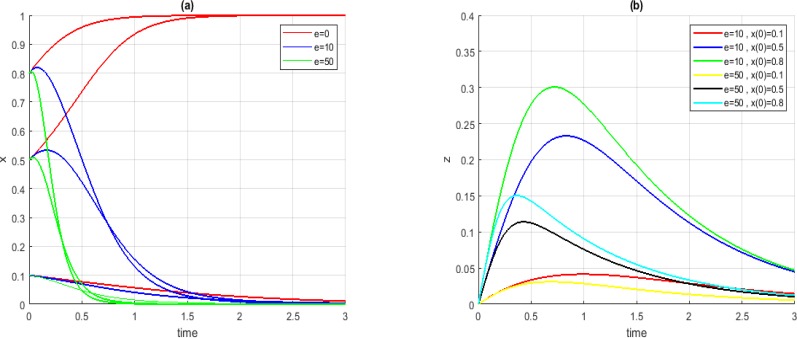
Convergence of *x* (a) and *z* (b) in Case *B_3_*, for *t_1_ = −1* and *t_2_ = +2*, different initial conditions and different values for *e*

Fig. 6 shows the system parameters convergence in Case *B_3_*. In this case, by choosing the parameter *e* in the interval described in Case *B_3_* the system would converge to value of 0 for the parameter *x*. However, by choosing higher amounts for *e*, the system would converge with a higher speed.

**Fig. 7:**
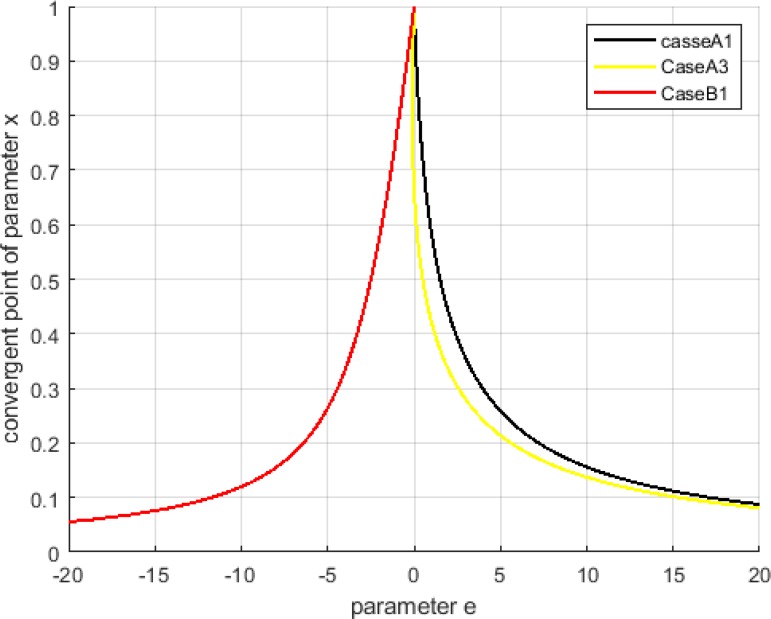
Steady state of the system for different amount of parameter *e*

Fig. 7 shows the steady-state of the system, which is convergence point of *x,* for different amounts of parameter *e*. The amount of parameters *t_1_* and t_*2*_ are the values shown in Figs. 3–6. This figure shows the effect of moderate changes in parameter *e* on the convergence of the system. Case *B_3_* is not discussed in this figure because its convergence depends on the initial conditions.

## Discussion

### Lack of Dataset

In our previous study, we modeled another aspect of cancer using game theory. Although we know that lack of validation using dataset is a drawback for our papers, in none of our works the models were validated based on real datasets because in each paper we discussed a specific aspect of cancer-based on biological concepts rather than whole cancer. In fact, our aim was to model different aspects of cancer using mathematical tools, a methodology that could contribute to eventual modeling of whole cancer.

## Applicability and Feasibility

How to apply our solutions to real patients? In this paper, the role of the immune system could be expressed by four parameters of the game (*a*, *b*, *c*, *d*). In fact, in the light of future studies, we would be able to determine the amount of these game parameters for each patient. The situation of each patient could be discussed as one of our separate cases.

How is it possible to change parameter *e* in reality? This parameter was based on three variables (*w*, *j*, *l*); so, by changing each of these variables, we can change the parameter *e*. Parameter *w* can not be changed easily, because it shows the effect of added stem cells on deduction of frequency of cancerous cells. On the other hand, *j* and *l* adjust the number of stem cells and we can regulate them. For instance, our suggested condition for case *A_1_* was *e = +∞*. Since it seems impossible to increase a parameter unboundedly, is it possible to operate our solutions in reality? To increase parameter *e*, we can increase parameter *j* or decrease parameter *l*, both of changed unboundedly. However, these extreme conditions are proposed to eradicate cancerous cells entirely, but Table 3 and result of the simulation section showed that with moderate changes in parameter *e*, the system can converge to the points with a low level of cancerous cells.

**Table 3:** Impact of gentle changes in parameter *e* on the equilibrium points of the system

	***t_1_***	***t_2_***	***e***	***X_eq2_***	***X_eq3_***
*A_1_*	−1	+2	0	1	2
			18	0.095	21
*A_3_*	1	2	0	0.66	1
			16	0.095	6.9
*B_1_*	−4	+1	0	−0.33	1
			−12	−3.4	0.097

Finding mathematical tools to model other aspects of cancer separately is a research topic proposition. Furthermore, new research can determine the values of our game parameters for given patients. Another suggestion for future studies is finding practical ways to change the game parameters to converge the system to the desired points. Also, an avenue for future works is to develop our analytical views towards other therapeutic methods, like chemotherapy and target therapy.

## Conclusion

The proposed model in this paper was based on improving the immune system response by adding stem cells to cure cancer. For the complete eradication of the cancerous cells, in some cases, severe conditions on stem cell-related parameters were required. Nevertheless, by applying less conservative requirements aligned with our suggestions, a large number of cancerous cells could be eradicated.

## Appendix

Here we prove [6].

From [2] and [3]:

$$\dot{x}=0\Rightarrow x(\mathrm{xa}+\mathrm{yb}-[x(\mathrm{xa}+\mathrm{yb})+y(\mathrm{xc}+\mathrm{yd})]\}=0$$

then, *x*{*xa* + (1 − *x*)*b* − [*x*(*xa* + *yb*) + *y*(*xc* + (1 − *x*)*d*)]} = 0

then, (−*a*+*b*+*c*−*d*)*x*^3^−(−*a*+2*b*+*c*−2*d*)*x*^2^+(*b*−*d*)*x*=0

then, (*t*_1_+*t*_2_)*x*^3^ − (*t*_1_+2*t*_2_)*x*^2^+*t*_2_*x*=0

where, *t*_1_ = *c* −*a*, *t*_2_ = *b* − *d*

then, *x*{(*t*_1_+*t*_2_)*x*^2^−(*t*_1_+2*t*_2_)*x*+*t*_2_}=0

then, *x*(*x*−1)[(*t*_1_+*t*_2_)*x*−*t*_2_]=0

then, *x*=0, *x*=1, $x=\frac{t_{2}}{t_{1}+t_{2}}$

Now, we prove our suggestion in (11). From (8) we have:

$$\Delta ={\left(t_{1}+t_{2}+e\right)}^{2}-4(t_{1}+t_{2})t_{2}$$

then:

$$\{\begin{array}{lll} if:e<R_{1} & \mathrm{then}:0<\Delta , & x_{\mathrm{eq}\;2,3}>0\\ \mathrm{if}:R_{1}<e<R_{2} & \mathrm{then}:0<\Delta & \\ \mathrm{if}:R_{1}<e & \mathrm{then}:0<\Delta , & x_{\mathrm{eq}\;2,3}<0 \end{array}$$

where, *R*_1,2_=±2(*t*_2_(*t*_1_+*t*_2_))^0.5^−(*t*_1_+2*t*_2_)
